# Placoderms and the evolutionary origin of teeth: a comment on Rücklin & Donoghue (2015)

**DOI:** 10.1098/rsbl.2016.0159

**Published:** 2016-09

**Authors:** Carole Burrow, Yuzhi Hu, Gavin Young

**Affiliations:** 1 Geosciences, Queensland Museum, 122 Gerler Road, Hendra 4011, Queensland, Australia; 2 Research School of Physics and Engineering (RSPE), Australian National University, Canberra 0200, Australian Capital Territory, Australia

## 1. Introduction

The extinct Devonian placoderms (armoured jawed fishes) [1,2] are central to the question of tooth origins, because some have denticulate ‘toothplates’ within the mouth cavity. A key question is whether these gnathal plates were modified from external dermal bones, or had ‘denticles’ representing true teeth with pulp cavities [3, fig. 2*h*]. The recent contribution by Rücklin & Donoghue [4] confuses this issue, because their claimed ‘anterior supragnathal’ (ASG) of the placoderm *Romundina stellina* shows no evidence that it came from the oral cavity, and is more likely an external dermal element. Also, the tissue identified as enameloid is not birefringent and thus not enameloid. Their inferences about growth of toothplates, phylogenetic loss of enameloid, and independent development of teeth and jaws, based on the structure of this plate, are therefore invalid.

## 2. Gnathal plate or dermal armour?

**Figure 1.**
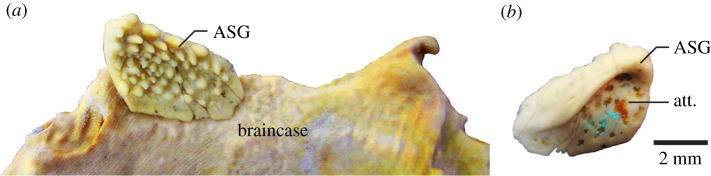
Early Devonian arthrodire ANU V244, specimen previously figured [8,9]: three-dimensional prototype of right anterior supragnathal (*a*) in position, ventral view; (*b*) depressed cancellous upper surface (att.) for braincase attachment. (Online version in colour.)

**Figure 2.**
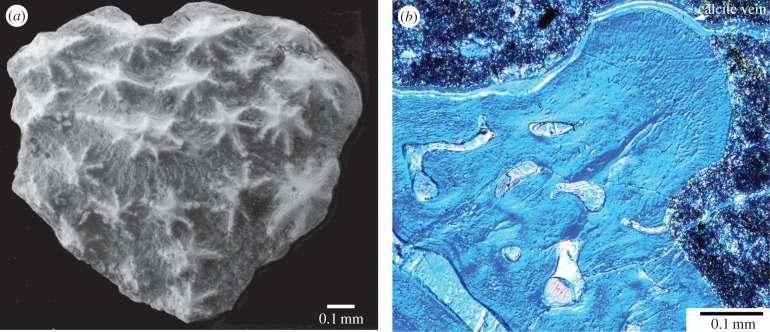
*Romundina* cf. *stellina* from *Romundina* type locality, Drake Bay Formation, Prince of Wales Island, Arctic Canada (Natural History Museum Paris collection). (*a*) Small plate DB4-95-1 [10, fig. 3*a*: ‘scale’]. (*b*) Vertical section through tubercle on spinal plate DB4-95-4 under crossed polars with DIC filters. (Online version in colour.)

The supposed ‘ASG’ came from “residues associated with the holotype of *R. stellina*” [4], but Ørvig [5] had asserted there were no gnathal elements in the type residues. Subsequent collections from the type locality contain numerous similar elements, and one articulated specimen with ASGs preserved in position [4, fig. 1*a*], as previously figured [6,7]. This ‘undetermined acanthothoracid’ [7] has the same dermal skull ornament of stellate tubercles as *Romundina* [5], but is a new taxon (cf. [4,6]) because the bone pattern is different. Its articulated ASGs have embayed posterior margins, and ornament of mainly elongate denticles with the smallest in the depressed central part [7, fig. 3*a*], representing the ossification centre as in typical supragnathal elements from the Early Devonian ([7–9]; figure 1). By contrast, the supposed ASG has convex margins [4], and the central (stellate) tubercle is largest and highest. Although it was claimed that “surface morphology of the tubercles … is quite distinct from … the dermal tubercles” [4], the latter are variable in *R. stellina* [5,10]; stellate tubercles on a typical small dermal plate (figure 2*a*) differ mainly from the supposed ASG in having more radiating ridges. We suggest the supposed ‘teeth’ are only dermal tubercles. Growth of the plate, by marginal addition without resorption, is normal for dermal platelets and scales [10, p. 207].

The supposed ASG was compared with the much younger (Late Devonian) derived arthrodire *Compagopiscis*, despite its different morphology [4, fig. 1*f–h*]. However, described Early Devonian arthrodire gnathals ([8,9]; not cited in [4]) all have a concave cancellous inner surface (figure 1*b*) for attachment to perichondral bone, completely unlike the convex lamellar bone inner surface on the supposed ASG [4]. External shape, tubercle type and overall morphology demonstrate that this element is not a gnathal bone; possibly it came from the mosaic of small ornamented plates in the *Romundina* ventral armour [7].

## 3. Histological interpretation

The tubercles, described as “multi-cuspid teeth, each composed of an enameloid cap and core of dentine” [4, p. 1], actually have enclosed cell spaces and no pulp cavity, thus demonstrating the special placoderm tissue ‘semidentine’, as in *Romundina* dermal tubercles ([5,11]; figure 2*b*). This histology is very different from typical tooth ‘orthodentine’, with no sign of the distinct pulp cavities seen in the derived arthrodire *Compagopiscis* [4, fig. 2*e*]. Also, the supposed enameloid layer, a zone densely filled with semidentine tubules perpendicular to the surface [11, fig. 41], shows no evidence of crystallites that would indicate enameloid; thin sections (figure 2*b*) show it is not birefringent. As enameloid cannot be demonstrated in *Romundina*, there is no support for the conclusion that enameloid was lost in other placoderms.

## Data accessibility

Data is available from the Dryad Repository (http://dx.doi.org/10.5061/dryad.9n237) [12].
